# Antioxidant Capacity and Total Phenolic Content in Fruit Tissues from Accessions of *Capsicum chinense* Jacq. (Habanero Pepper) at Different Stages of Ripening

**DOI:** 10.1155/2014/809073

**Published:** 2014-02-11

**Authors:** Lizbeth A. Castro-Concha, Jemina Tuyub-Che, Angel Moo-Mukul, Felipe A. Vazquez-Flota, Maria L. Miranda-Ham

**Affiliations:** Unidad de Bioquímica y Biología Molecular de Plantas, Centro de Investigación Científica de Yucatán, Calle 43 No. 130, Chuburná de Hidalgo, 97200 Mérida, YUC, Mexico

## Abstract

In the past few years, there has been a renewed interest in studying a wide variety of food products that show beneficial effects on human health. *Capsicum* is an important agricultural crop, not only because its economic importance, but also for the nutritional values of its pods, mainly due to the fact that they are an excellent source of antioxidant compounds, and also of specific constituents such as the pungent capsaicinoids localized in the placental tissue. This current study was designed to evaluate the antioxidant capacity and total phenolic contents from fruits tissues of two *Capsicum chinense* accessions, namely, Chak k'an-iik (orange) and MR8H (red), at contrasting maturation stages. Results showed that red immature placental tissue, with a Trolox equivalent antioxidant capacity (TEAC) value of 55.59 **μ**mols TE g^−1^ FW, exhibited the strongest total antioxidant capacity using both the 2,2-diphenyl-1-picrylhydrazyl (DPPH) and the CUPRAC methods. Placental tissue also had the highest total phenolic content (27 g GAE 100 g^−1^ FW). The antioxidant capacity of *Capsicum* was directly related to the total amount of phenolic compounds detected. In particular, placentas had high levels of capsaicinoids, which might be the principal responsible for their strong antioxidant activities.

## 1. Introduction

Chili peppers (*Capsicum spp.*) are among the most consumed vegetables in the world. Mexico is considered an important center of domestication and diversification of chili peppers. Given the extension of the territory and variations in environmental conditions, the different regions have developed their own cultivars and varieties. For instance, although in the Yucatan Peninsula indigenous cultivars of *C. annuum* have arisen, Habanero peppers (*C. chinense*) are widely preferred and an extensive variety of cultivars is available [1]. Habanero peppers from Yucatan include varieties that differ in color, size, and capsaicinoid content.

Even though the main attribute of peppers is its pungency that results from the presence of capsaicinoids, they are also highly valued as an excellent source of natural pigments and antioxidant compounds. The main edible part of the fruit, the pericarp, contains high amounts of ascorbic acid, vitamins A and E, carotenoids, and phenolic compounds [2, 3], which are considered strong antioxidants. Oxidative stress has been related to damages provoked by aging and various ailments in humans [4]. Thus, the intake of foods rich in antioxidants is recommended to prevent such disorders [5, 6]. In fact, there is an increased interest in the food industry to use fortified plants or functional ingredients [7, 8]. Functional ingredients mainly correspond to phytochemicals, and given their complex reactivity, the antioxidant power of plant extracts cannot be properly evaluated by a single method. Therefore, at least two different tests are recommended for the proper determination of antioxidant activity in foodstuff; one for the free radical-scavenging activity and another for the total antioxidant capacity [9]. Here, we evaluated the antioxidant capacity of two accessions of Habanero peppers cultivated in the Yucatan Peninsula differing in the color of the ripe pericarp. Since peppers are mainly consumed for their pungency, which is due to their capsaicin content, and this compound is produced and accumulated in the placental tissue, peppers were dissected into pericarps and placentas, and each tissue was analyzed independently. Moreover, the antioxidant activity was evaluated using 1,1-diphenyl-2-picrylhydrazyl (DPPH) radical and the cupric-reducing antioxidant power (CUPRAC) assays. In order to complement these determinations, total phenolic contents were also quantified.

## 2. Materials and Methods

### 2.1. Plant Material

Pods from two local accessions of habanero pepper, Chak k'an-iik (orange pericarp) and MR8H (red pericarp), from plants cultivated in a greenhouse at the Centro de Investigación Científica de Yucatán, were collected at two different physiological stages: immature (green pericarps; approximately 25 days postanthesis (DPA)) and fully mature (orange or red color pericarps; 40 DPA). Pods were washed with commercial soap and thoroughly rinsed with running tap water before pericarps and placental tissues were delicately separated. Tissues were weighed separately, then frozen with liquid nitrogen, and stored at −80°C until analyzed.

### 2.2. Extraction Procedure

Tissues were extracted as described in [10], with some modifications. Approximately 1 g FW was ground with liquid nitrogen and extracted with 5 mL 80% ethanol. The homogenate was centrifuged at 2600 ×g for 30 min at 4°C and the clear supernatant was collected and stored at 4°C until analysis.

### 2.3. Assays for Antioxidant Activity

#### 2.3.1. DPPH Radical-Scavenging Activity

Free radical-scavenging activity was evaluated using a modified DPPH assay [11]. DPPH was freshly prepared in 96% ethanol and an aliquot from a 0.4 mM solution was mixed with 100 mM Tris-HCl, pH 7.4, and different volumes of antioxidant samples to a final volume of 2 mL. Mixtures were vigorously shaken and let to stand at room temperature for 30 min. Absorbance was recorded at 517 nm and % of inhibition of free radical was calculated as [(*A*_DPPH_ − *A*_*S*_)/*A*_DPPH_] × 100, where *A*_DPPH_ is the absorbance of the control reaction (containing all reagents without the extract) and *A*_*S*_ is the absorbance of the extract. Extract concentration inducing 50% inhibition (IC_50_) was calculated from the graph (slope = −0.0171) of radical scavenging activity percentage versus sample extract. Trolox (6-hydroxy,2,5,7,8-tetramethylchroman-2-carboxylic acid) was used as a positive control [12, 13].

#### 2.3.2. Trolox Equivalent Antioxidant Capacity (TEAC)

The antioxidant capacity was assayed as the reaction of DPPH with Trolox, as described by Oomah et al. [14]. Pericarps (10–60 *μ*L) or placentas (5–15 *μ*L) extracts were mixed with 1 mL 0.4 mM DPPH dilution in Tris buffer (100 mM, pH 7.4), vigorously shaken and allowed to reach a steady state at room temperature. A standard curve (0 to 60 *μ*M) was prepared using Trolox solutions. The decrease in absorbance at 520 nm 30 min after addition of a compound was used for calculating the TEAC. All determinations were performed in triplicate. Results were expressed as *μ*moles Trolox equivalent (TE) g^−1^ FW [15, 16].

#### 2.3.3. Total Phenolic Content

Total polyphenols in extracts were determined [17], using the Folin-Ciocalteu reagent and gallic acid in methanol (0–0.5 mg mL^−1^) as a standard. Briefly, 0.02 mL of extract and 1.58 mL water were mixed with 0.1 mL of the Folin-Ciocalteu's reagent, followed by 0.3 mL 20% (w/v) sodium carbonate. After 2 h at room temperature, absorbance was measured at 765 nm. Total phenolic content was calculated as gallic acid equivalents (GAE) 100 g^−1^ FW.

#### 2.3.4. CUPRAC Total Antioxidant Capacity Assay

Cupric reducing antioxidant power (CUPRAC) was determined as described in [18]. Equal volumes (1 mL) of 100 mM CuCl_2_, 7.5 mM neocuproine alcoholic solution, and 1 M ammonium acetate buffer were mixed prior to addition of plant extracts and water to a final volume of 4.1 mL in a tube. Tubes were thoroughly mixed and let to stand for 30 min before absorbance at 450 nm was measured [19, 20].

### 2.4. Statistical Analysis

Data were expressed as means ± SD. Statistical analysis was performed by using a one-way ANOVA statistical model and mean comparisons were made using Tukey's multiple-range test at a 5% level of probability.

## 3. Results and Discussion

### 3.1. Free Radical Scavenging Activity DPPH of *Capsicum* Fruits

In order to obtain a complete prospect of the antioxidant potential of Habanero peppers, assays to evaluate radical scavenging and total reducing power were carried out.

Free radical scavenging activity was evaluated using DPPH, which estimates antioxidant capacity as IC_50_ and TEAC. IC_50_ represents the dose of sample that reduces by half the absorbance of a DPPH reference solution; therefore, a low IC_50_ indicates a high antioxidant activity [21]. The lowest radical scavenging activity (highest IC_50_ values) was found in the immature, rather than mature pericarps of both *C. chinense* accessions (Table 1). These differences were not observed for placentas (Table 1).

In contrast to Habanero, in bell peppers, immature pericarps, regardless of their final coloration, presented higher antioxidant capacity than the ripe state [22]. These results suggest that the composition of pericarps in these two species does not follow similar patterns through the maturation process. It is interesting to notice that placental tissues possessed a noticeable higher antioxidant capacity than pericarps (*ca*. 10-fold in both varieties; Table 1).

Capsaicinoids are present only in the *Capsicum* genus and confined to the epidermal cells of the placental tissue [23]. They present antioxidant properties similar to those of flavonoids [24].

These results were confirmed when antioxidant potential was expressed as TEAC; that is, placental tissues exhibited a higher antioxidant capacity compared to pericarps (Table 2). It is noteworthy to point out that red pod placentas had in average up to 10-fold higher antioxidant capacity than pericarps from the two accessions (Table 2).

### 3.2. Total Phenolic Content in Habanero Peppers

**Figure 1:**
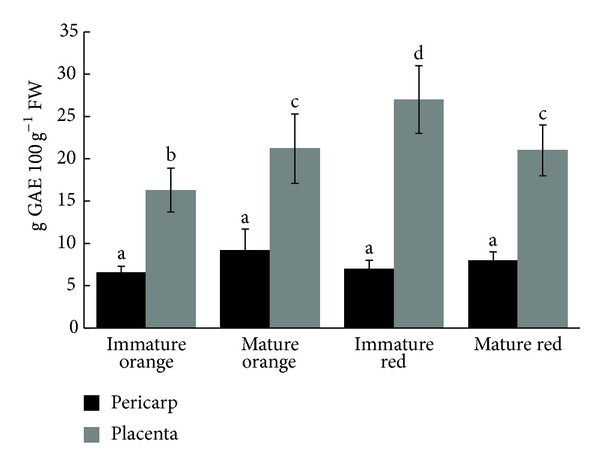
Total phenolic contents in two habanero cultivars at different maturation stages. Values are mean ± SEM, *n* ≥ 3. Values with distinct letters differ significantly (*P* < 0.5).

No significant differences were found regarding total phenolic contents in the pericarps from the two accessions, which presented lower levels in comparison to placental tissues, regardless of their maturation stage. The highest contents were found in placentas from red peppers (Figure 1), reaching a value of 27 g GAE 100 g^−1^ FW, which represents a 150% increase over the amount found in pericarps.

It should be noted that Habanero peppers present higher phenolic contents than strawberries [25], tomatoes [26], or *C. annuum *red pods [21]. Phenolics contents in Habanero were 80-fold higher than in Serrano peppers if only the placental levels are considered [27]. However, when these were presented per pod, levels in Habanero were even higher (112-fold).

Interestingly, the maturation stage had no effect on the phenolic contents neither in pericarps nor in placentas, regardless of the accession analyzed. Data differ from those found in *C. annuum* fruits [28] and in *C. annuum*, *C. frutescens*, and *C. chinense* [29] where the phenolic content varies in relation to the degree of ripeness.

The high levels of phenolic compounds found in Habanero pepper pods could well be associated to both capsaicinoids and the intermediaries in their biosynthetic pathway, such as coumaric acid, caffeic acid, and ferulic acid [30].

### 3.3. Total CUPRAC Antioxidant Activity

**Figure 2:**
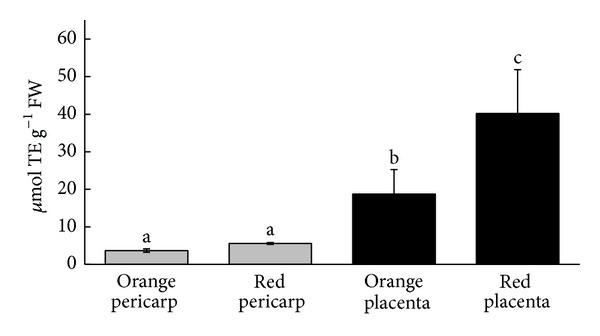
Total CUPRAC antioxidant activity in pericarps and placentas from two immature *C. chinense* Jacq. accessions. Values are mean ± SEM, *n* ≥ 3. Differences are significant at level *P* < 0.05.

Antioxidant capacity of pod extracts was also determined using the CUPRAC assay. Similar values (*μ*moles TE g^−1^ FW) were found in pericarps from immature pods of both varieties. In contrast, the placental tissues presented between three- (orange) and eight- (red) fold higher phenolic contents than pericarps (Figure 2).

**Figure 3:**
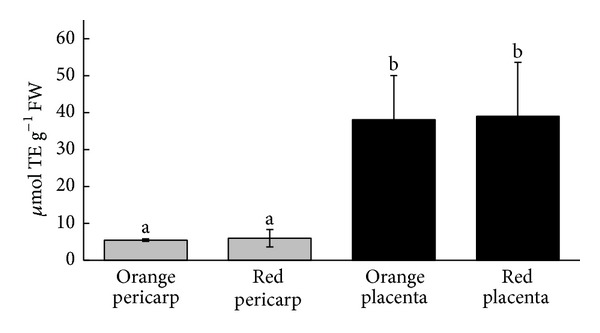
Total CUPRAC antioxidant activity in pericarps and placentas of two ripe *C. chinense *Jacq. accessions. Values are mean ± SEM, *n* ≥ 3. Differences are significant at level *P* < 0.05.

When the mature pods were used, no differences could be found between accessions regarding the pericarps or the placentas' antioxidant capacity; however, placental tissues presented ca. of 40 *μ*moles TE g^−1^ FW, which represent values 10-fold higher than those in the pericarps (Figure 3).

Considering that high levels of phenolics were contained in tissues with also high antioxidant activity, it is proposed that an important component of this activity in the *C. chinense* accessions could be ascribed to such phytochemicals. In fact, antioxidant activity presented linear relationships to their total phenolic contents (*r* ^2^ = 0.9843).

In conclusion using different assays to determine a full prospect of the total antioxidant capacity, it was determined that the *C. chinense* Jacq. accessions from the Yucatan Peninsula have significant amounts of antioxidants, specifically in the placental tissue. The antioxidant properties of the Habanero pepper may be explained in terms of the phytochemicals that it contains, such as carotenoids, vitamins, phenolic compounds, and capsaicinoids [3, 31–33]. Human consumption of *C. chinense* pods may supply a substantial amount of the antioxidants needed to promote a better health and to prevent diseases and ailments. Our results can be the basis for the selection of suitable accessions for antimicrobial and anticarcinogenic trials of habanero extracts.

## Figures and Tables

**Table 1:** IC_50_ values of pericarps and placentas from two *C. chinense* Jacq. accessions at different maturation stages.

IC_50_ (µL)		
	Habanero orange	Habanero red
Immature pericarp	128.73 ± 11.09	131.56 ± 14.06
Mature pericarp	52.2 ± 4.81	71.94 ± 2.68
Immature placenta	17.10 ± 6.70	10.12 ± 1.15
Mature placenta	15.95 ± 7.42	10.75 ± 0.56

Values represent mean (*n* = 3) ± SD.

**Table 2:** TEAC antioxidant capacity of *C. chinense *ethanolic extracts.

TEAC (µmols TE g^−1^ FW)		
	Habanero Orange	Habanero Red
Immature pericarp	4.17 ± 0.55	4.22 ± 0.82
Mature pericarp	8.84 ± 1.77	6.67 ± 1.03
Immature placenta	30.08 ± 6.99	55.59 ± 8.60
Mature placenta	41.64 ± 4.01	42.28 ± 6.99

Values represent mean (*n* = 3) ± SD.
